# Microfluidic Devices Developed for and Inspired by Thermotaxis and Chemotaxis

**DOI:** 10.3390/mi9040149

**Published:** 2018-03-26

**Authors:** Alireza Karbalaei, Hyoung Jin Cho

**Affiliations:** Department of Mechanical and Aerospace Engineering, University of Central Florida, Orlando, FL 32816, USA; akarbalaei@knights.ucf.edu

**Keywords:** thermotaxis, chemotaxis, microfluidic, taxis, thermocapillary, chemocapillary, bio-inspired, microorganism

## Abstract

Taxis has been reported in many cells and microorganisms, due to their tendency to migrate toward favorable physical situations and avoid damage and death. Thermotaxis and chemotaxis are two of the major types of taxis that naturally occur on a daily basis. Understanding the details of the thermo- and chemotactic behavioral response of cells and microorganisms is necessary to reveal the body function, diagnosing diseases and developing therapeutic treatments. Considering the length-scale and range of effectiveness of these phenomena, advances in microfluidics have facilitated taxis experiments and enhanced the precision of controlling and capturing microscale samples. Microfabrication of fluidic chips could bridge the gap between in vitro and in situ biological assays, specifically in taxis experiments. Numerous efforts have been made to develop, fabricate and implement novel microchips to conduct taxis experiments and increase the accuracy of the results. The concepts originated from thermo- and chemotaxis, inspired novel ideas applicable to microfluidics as well, more specifically, thermocapillarity and chemocapillarity (or solutocapillarity) for the manipulation of single- and multi-phase fluid flows in microscale and fluidic control elements such as valves, pumps, mixers, traps, etc. This paper starts with a brief biological overview of the concept of thermo- and chemotaxis followed by the most recent developments in microchips used for thermo- and chemotaxis experiments. The last section of this review focuses on the microfluidic devices inspired by the concept of thermo- and chemotaxis. Various microfluidic devices that have either been used for, or inspired by thermo- and chemotaxis are reviewed categorically.

## 1. Introduction

Cells, nematodes, bacteria and many other microorganisms have the ability to sense the physical changes in their surroundings and move toward the more favorable situations and avoid damage or death. Based on the nature of microorganisms’ response, different categories of biological responses to environmental stimulations are defined, such as kinesis [1], taxis [2] and tropism [3]. Tropism is categorized as a growth response, and kinesis is defined as the undirected locomotory reaction of cells and organisms [4,5]. Taxis, however, is the directional migration of cells and microorganisms in response to a stimulation, various forms of which have been seen in nature. The detail of the response is different in taxis based on the various types of stimulus. Several types of taxis are known today among which thermotaxis [6], chemotaxis [6], rheotaxis [7], aerotaxis [8], phonotaxis [9] and phototaxis [10] can be mentioned. Taxis is categorized as positive and negative depending on whether the animals are attracted to or repelled by the stimulus [11]. Accurate understanding of sample cells and microorganisms such as nematodes, bacteria and sperm is crucial in the screening of mutants, exploring the relationship between neural circuit functions and behavior and exploring therapeutic drugs for neurodegenerative diseases such as Parkinson’s disease, Alzheimer’s disease and autism spectrum disorder [12,13].

**Figure 1:**
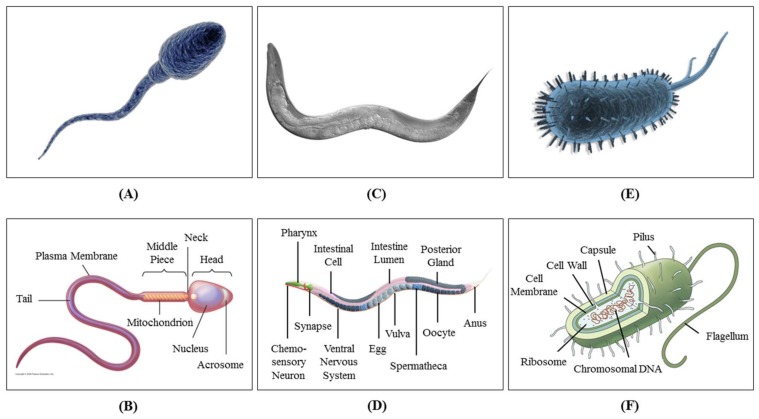
(**A**) Schematic 3D picture of a sperm (Photo Credit: iStockphoto, modified). (**B**) Different parts of a sperm (Copyright © 2009 Pearson Education, Inc.). (**C**) DIC Nomarski image (collage of multiple shots) of *C. elegans* N2 (wildtype) adult hermaphrodite (© Ian D. Chin-Sang, 2017). (**D**) Different parts of a *C. elegans* (photo credit is given to BodyFIG1B, and the name of the organs are from the work by Kaletta and Hengarter [17]). (**E**) Schematic 3D picture of the bacterium called *Escherichia coli* or *E. coli* (Photo Credit: 3DOcean, modified). (**F**) Different parts of an *E. coli* (Photo Credit: emaze.com).

The term is thermotaxis if the cause of stimulation is a thermal gradient. Cells and microorganisms are very sensitive to temperature change, and monitoring their behavioral response is necessary for clinical diagnostic and treatment purposes. Cells, nematodes, bacteria and sperm often have certain living and reproduction ranges of temperature that they seek. Thermotactic response is their defensive mechanism to migrate toward the desired temperature and avoid temperatures that trigger undesired reactions in them, causing damage or death. The thermotaxis behavior of several different cells and microorganisms has been analyzed so far, among which spermatozoon [14], *Caenorhabditis elegans* or *C. elegans* (from the family of nematodes) [15] and *Escherichia coli* or *E. coli* (from the family of bacteria) [16] have been the most common due to their sensitive response and vast applications. Figure 1 shows what these organisms look like. The study of thermotaxis helps researchers to understand body behavior at the neuronal level and to eventually be able to diagnose and cure neurodegenerative diseases.

**Figure 2:**
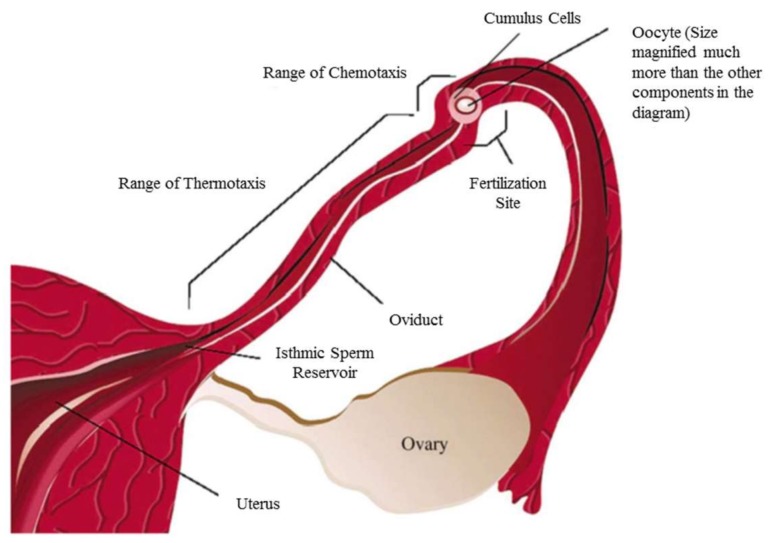
Sperm guidance mechanism in mammalian female genital tract, which shows the relative range of action of thermotaxis and chemotaxis. Reprinted from [18].

Chemotaxis, however, refers to the directional response of cells, nematodes and bacteria to a particular chemical composition. One of the differences between thermo- and chemotaxis is their range of effectiveness. It is known that thermotaxis is a rather long-range guidance mechanism for sperm, while the effective distance in chemotaxis is much shorter, as is shown in Figure 2 [18]. Each individual cell or microorganism can be attracted toward or repelled by certain chemical gradients. One of the apparent chemoattractants of any living organism is its food source [19]. The point of chemotaxis (just like thermotaxis) is the smart movement of cells and microorganisms toward the favorable region to avoid a fatal situation. The study of the chemotactic response of cells and microorganisms reveals their reaction to different chemicals in terms of quality and speed and has applications in drug delivery, as well as enhancing diagnostics and curing of degenerative, infectious and inflammatory diseases [20,21,22,23].

From the mechanical point of view, taxis in different forms has inspired the development of microfluidic devices. In general, any physical characteristics that are functions of temperature and chemical composition of the surrounding environment can be used for fluid manipulation. One of these physical characteristics in solid/liquid or liquid/liquid interactions is surface tension, whose change with temperature and chemical composition has been used to induce fluid flow. Therefore, inspired by thermo- and chemotaxis, thermal or chemical gradients have been imposed and utilized in microfluidic systems to induce gradients in surface tension and generate liquid mass transfer. This phenomenon is called thermo-/chemocapillarity, and the shear flow induced by it has been used to enhance liquid mixing, migration and evaporation, as well as the fabrication of fluidic valves, pumps, traps, etc.

In this article, thermotaxis and chemotaxis, as two of the most common forms of taxis, are reviewed. At the beginning, a brief biological overview is presented about these two forms of taxis. The rest of the review is devoted to microfluidic devices used for thermo- and chemotaxis, as well as devices inspired by them. In the last section, a summary is provided, and microfluidics-related literature on thermo- and chemotaxis devices are categorized in a table.

## 2. Biological Overview

### 2.1. Thermotaxis

The reaction of body organisms to temperature variations is of crucial importance since they are commonly very sensitive to the slightest temperature deviation from normal [24]. This is why an imbalance in body temperature is one of the most common symptoms in most diseases, and its restoration to normal is considered as one of the therapeutic signs. The term “thermotaxis” was first used in medicine in 1890 to address thermal regulations inside the body [25]. Later, the concept was used in microbiology to manipulate organisms by means of a temperature gradient. Thermotactic behavior of cells and microorganisms is divided into positive and negative responses, thermophilic and cryophilic locomotion, respectively [26]. During positive thermotaxis, the cells are attracted to the higher temperature, while in negative thermotaxis, they are repelled by warmth. This phenomenon is used in neuroscience to analyze the behavioral response of sensory neurons, interneurons and motor neurons toward changes in temperature [27]. Sample organisms such as nematodes, bacteria, sperm and different kinds of larvae have been analyzed for their thermotactic behavior.

#### 2.1.1. Thermotaxis of Nematodes

Nematodes have been under study for their thermotactic behavior since 1960, when Parker and Haley published their results for thermotaxis analysis of *Nippostrongylus muris* (*N. muris*) [28]. In 1969, El-Sherif and Mai ran experiments on the thermotaxis of three plant parasitic nematodes, namely *Ditylenchus dipsaci* (*D. dipsaci*), *Pratylenchus penetrans* (*P. penetrans*) and *Tylenchorhynchus claytoni* (*T. claytoni*) [29]. *C. elegans* is known as the most common nematode sample for thermotaxis analysis due to its interesting thermotactic response and its analogy to neural cell behavior. *C. elegans* is a free-living transparent nematode that lives in a soil environment. In 1975, a research group from Caltech studied the thermotaxis behavior of this nematode by culturing it at different temperatures ranging from 16 °C–25 °C and then placing it in a thermal gradient [30]. They found out that it migrates toward its original cultivation temperature and then moves isothermally. The response time and thermotaxis defects were studied on different asynchronous adult mutants, and this thermotactic behavior was captured and analyzed using different imaging techniques under spaceflight conditions [31]. In 2005, Ito et al. analyzed the thermotaxis behavior of *C. elegans* by the quantitative population thermotaxis assay using a gentle thermal gradient to resolve inconsistencies in the previous studies [32]. While some of the recent work at the time proposed that the thermotactic behavior of *C. elegans* is stochastic, Ito and his coworkers proved that the earlier models of the two-step migration of *C. elegans* are more accurate at least for nematodes cultivated at lower temperatures. This means that thermotaxis in these nematodes is regulated by the counterbalance between two opposite phenotypes, namely thermophilic and cryophilic. It is translated as a preference of migration toward the cultivation temperature of animals, which then move isothermally perpendicular to the temperature gradient. In none of the thermotaxis studies was a clear migration trend captured for *C. elegans* cultivated at temperatures above 20 °C; therefore, their thermotactic behavior is described as stochastic. However, the ones that have been cultivated at lower temperatures still follow the two-step migration function. According to Ito et al. [32], this inconsistency can be addressed by analyzing the nematodes for a longer period of time after they are placed in the thermal gradient. This is due to the fact that the response time for the ones cultivated at higher temperatures is greater than average (at least around 30 min). Moreover, correlating the nematodes’ response with temperature change has been proven to increase the sensitivity of detection. Numerous attempts have been made by different research groups for the analysis of the thermotaxis response of *C. elegans*, to which interested readers are referred for more detailed discussions [33,34,35,36,37,38,39,40,41].

#### 2.1.2. Thermotaxis of Bacteria

Research on bacterial thermotaxis also started to develop around the 1970s. Imae and his group at Nagoya University were among the pioneers in this field. They published the temperature effect on the motility and chemotaxis of *E. coli* in 1976 [42]. They used L-serine to prove that the thermosensory behavior of *E. coli* is interconnected with its chemosensory behavior so that adding the chemical reduces thermosensitivity in the bacteria [43]. Later, it was reported that thermotactic behavior in *E. coli* is in fact bidirectional depending on the chemical conditions of its surroundings [44,45]. Cryophilic locomotion of *E. coli* has been reported, although the temperature gradient has to be greater than 0.02 °C/μm to be able to capture this response. In situations where the thermal gradient is weaker than the threshold, thermophilic behavior is observed [46,47,48]. Numerous other experimental analyses have been performed on the thermotaxis of *E. coli* under various environmental situations to reveal the details of their bidirectional response [49,50,51].

#### 2.1.3. Thermotaxis of Sperm

Sperm thermotaxis has been under researchers’ attention since 2003, when Bahat and her group proposed thermotaxis as a potential navigation mechanism for sperm in the female genital tract [14]. It is important to understand the exact and detailed behavior of sperm from the moment they start migrating from the male genital tract and swim through the female genital tract toward the egg. Numerous efforts have been made to mimic this journey on-chip and reveal various migration behaviors [52,53]. For example, it is reported that the temperature distribution inside the female genital tract is different during ovulation, which affects the migration characteristics of sperm [14]. Accurate quantitative analysis of thermotactic motion of sperm under various temperature gradients over certain periods of time has also been performed [54]. At this point, researchers have succeeded in capturing this thermotactic migration at minuscule temperature gradients as small as only 10^−5^ °C/μm [55,56].

### 2.2. Chemotaxis

Migration of cells and microorganisms toward optimum chemical conditions, either to seek an energy source or to avoid fatal situations, is called chemotaxis [57]. According to Keller and Segel [58], the chemotactic response of these organisms is analogous to Brownian motion. This means that, despite migration fluctuations of each single cell, the averaged motion of many cells leads to a macroscopic flux, which is proportional to the chemical gradient. The concentration of certain chemicals has also been reported to have a role in the determination of other types of tactical behavioral patterns of cells and microorganisms. The chemotactic response of different cells and microorganisms, such as nematodes, bacteria, leukocytes and sperm, is discussed in this review, although numerous valuable studies have been done on others such as blood cells [59], as well. 

#### 2.2.1. Chemotaxis of Bacteria

The *E. coli* bacterium is one of the most common bacteria whose chemotactic response has been studied so far. Adler stated in 1966 that *E. coli* is chemotactic toward oxygen and other energy sources such as galactose, glucose, aspartic acid, threonine and serine [57]. He also examined the chemotactic response of *E. coli* to amino acids in the same setup in a separate study [60]. In previous studies, avoidance of high concentration regions was observed after bacteria reached the maximum level of the energy source, which was a counterintuitive behavior. In the analysis of the chemotactic behavior of mutant *E. coli* K12 in response to amino acids such as serine and aspartate, in 1972, Berg and Brown showed that the migration of bacteria toward the lower concentration after reaching the highest concentration of energy source is part of its neutral and random movement and can be considered as only minimally important [61].

#### 2.2.2. Chemotaxis of Leukocytes

A special group of cells whose chemotactic response has been of interest is leukocytes. A Boyden Millipore system was employed by Zigmond and Hirsch in 1972 to analyze the chemotactic behavior of polymorphonuclear leukocytes (PMNs) [62]. They addressed the difference between chemotaxis of PMNs and stimulation of them through inducing random locomotion and immobilizing them in certain regions with a higher concentration of chemoattractants. In 1975, Wilkinson proposed the possibility of modifying the chemotactic response pattern of leukocytes using lipid-specific toxins such as oxygen-labile lysins (streptolysin 0 and clostridium perfringens θ, for example) [63]. Later, Forrester and Wilkinson analyzed the effect of hyaluronic acid on the motility of neutrophil leukocytes using the same system, in vitro [64]. In an article published in the Proceedings of the National Academy of Sciences in 1987, transforming growth factor type β (TGF-β) as an immunoregulatory peptide was used as a potential chemoattractant for human peripheral blood monocytes between concentrations of 0.004 and 0.4 pM [65]. The existence of high-affinity receptors on the surface of this cell, which are very sensitive to TGF-β ligands at low concentrations, makes this ligand potentially the best chemoattractant for blood monocytes. There are numerous review papers on this subject to which readers are referred for more detailed discussions [66,67,68]. Moreover, there is a book published on the subject of leukocyte locomotion and chemotaxis including 26 papers from the first international conference held on the matter in Gersau, Switzerland, in 1982 [69].

#### 2.2.3. Chemotaxis of Sperm

Sperm is one of the other groups of cells whose chemotactic response has been of interest. In 1951, during a set of statistical experiments on rat semen, Moricard and Bossu reported that spermatozoa migrate toward the oocyte at the time of fertilization [70]. Around the same time, Perloff [71] and Schuartz et al. [72] also observed chemotactic locomotion of sperm toward ovarian cyst fluid, in separate studies. It has also been proven that progesterone, which is secreted by cumulus cells after ovulation, is a major chemoattractant for sperm [73]. Agarwal divides sperm chemotactic response into two steps: first, there is a change of orientation toward the source of progesterone, and then, there is swimming to the high concentration region. The chemotactic behavior of bracken spermatozoa in response to malic acid was studied [74] as an example of chemotaxis in plant reproduction. It has also been reported that sperm of *Campanularia flexuosa* and *Campanularia calceolifera* shows pre-fertilization chemotactic behavior toward female gonangium by accumulating around its aperture [75]. 

## 3. Microfluidic Devices for Taxis Experiments

Considering the length-scale and range of effectiveness in these phenomena, advances in microfluidics have facilitated taxis experiments and enhanced the precision of controlling and capturing the microscale samples. Microfabrication of fluidic chips could bridge the gap between in vitro and in situ biological assays, specifically in taxis experiments. On-chip studies of the taxis response of cells and microorganisms can be divided into high-throughput and single-cell studies. During a high-throughput study, several cells are injected into the device as a batch and are treated to the stimuli simultaneously. However, in a single-cell neural levels study, the quality of the response of a single cell or microorganism toward a specific kind of stimulus is important [76,77]. For the latter group of experiments, it is often beneficial to hold the sample stationary to reduce uncertainties since the frequency and nature of encounters are considered uncontrolled experimental factors in free-moving experiments.

### 3.1. Thermotaxis

**Figure 3:**
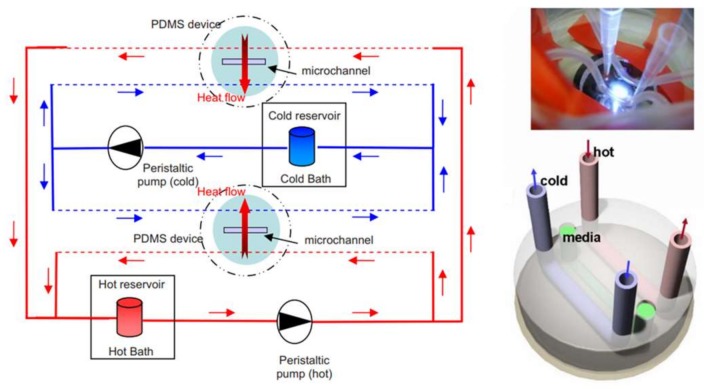
Using the counterflow heat exchanger concept at the microscale for the generation of a constant temperature gradient across a microchannel for thermotaxis experiments on-chip. Reproduced from [79].

Microfluidic-based thermotaxis is a younger field and has more potential for progress compared to chemotaxis. Devices have been fabricated for thermotaxis of some cells and microorganisms such as sperm, nematodes and bacteria. However, no microfluidic device for thermotaxis of blood cells such as leukocytes has been developed yet, although their thermotactic response was reported years ago [78]. Thermotaxis in microfluidics can be conducted using microfluidic thermal gradient systems (μTGS) in which a thermal gradient is generated by a counterflow heat exchanger, as shown in Figure 3 [79]. The main advantage of these systems is eliminating joule heating and the need for metal elements.

#### 3.1.1. Microfluidics for Sperm Thermotaxis

**Figure 4:**
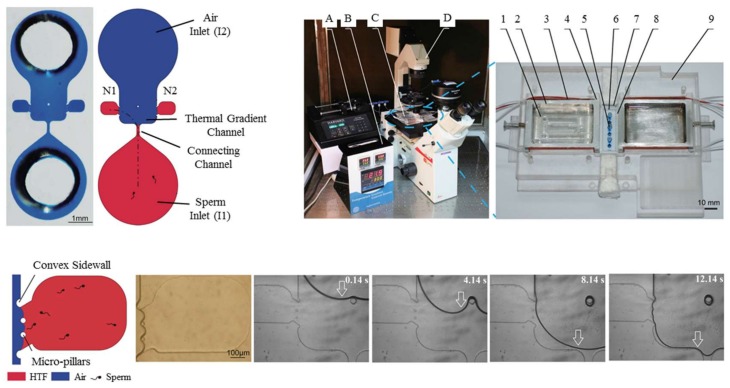
Microfluidic device along with the experimental apparatus used by Li et al. to perform sperm thermotaxis. The device is comprised of two inlets, one for sperm samples and the other for air. Adjusting the air pressure can stop the swimming of sperm toward collection reservoirs N1 and N2 by the help of microposts. The parts in the experimental apparatus are: A, syringe pump; B, digital temperature controller; C, temperature gradient generator; D, inverted microscope. Different sections of the microfluidic device are: 1, glycerol; 2, aluminum alloy tank; 3, resistive heater; 4, thermistor; 5, microfluidic channel; 6, PDMS upper layer; 7, glass lower layer; 8, chip positioning chamber; 9, PMMA case. Reproduced from [80].

In 2014, Li et al. designed and tested a PDMS (polydimethylsiloxane) microfluidic device demonstrating the thermotaxis phenomenon on human sperm manipulation [80]. As is shown in Figure 4, their device is comprised of two inlets, one for the sample and another for air. Thermotaxis of sperm is demonstrated by manipulating them toward either of the two collecting reservoirs by imposing different intensities of the lateral thermal gradient. By changing the air pressure at the inlet, the interface is programmed to progress toward the connecting channel causing the sperm to stop swimming toward the collecting reservoirs, hence working as a valve. Figure 4 shows their microfluidic device and experimental platform, as well as the temperature gradient profile.

In this study, the thermotaxis index (TI) is defined as a factor to analyze this behavior. The thermotaxis index is defined as the ratio of the number of sperm migrating to the warm reservoir to the number of sperm travelling to the cold reservoir. According to this definition, the response of the sperms to the temperature gradient is positive thermotaxis if TI > 1.0 and is negative thermotaxis if TI < 1.0. After TI is determined for a control group, the number of cells (in this case, sperms) migrating by thermotaxis can be compared to the total quantity of the control group to define the thermotaxis percentage, TP, in order to measure the strength of the thermotactic response of the organisms. In the work by Li et al. [80], TP gets as high as around 11%, and TI is greater than 1.0.

#### 3.1.2. Microfluidics for Nematodes Thermotaxis

**Figure 5:**
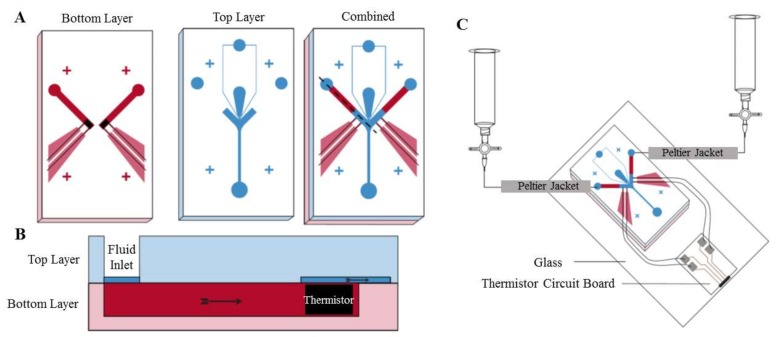
Microfluidic chip enhanced with fluidic tweezers fabricated by McCormick et al. used for thermotaxis analysis of *C. elegans*. Reprinted with permission from [81].

McCormick and his coworkers used a pair of microfluidic tweezers in their study of the thermotaxis effect on *C. elegans* [81] (Figure 5). Using this technique, they succeeded in analyzing the behavior of a single nematode in a stationary situation and reduced uncertainties. They concluded that this response is very similar to klinotaxis, which is the wavering side-to-side motion of the head of an organism in response to a stimulus as the result of continuous sampling of single receptors to determine the direction of movement. Furthermore, thermotaxis down the gradient is more favorable for this animal, which is consistent with previous studies. They used the same approach to analyze the chemotactic response of *C. elegans*, as well.

#### 3.1.3. Microfluidics for Bacteria Thermotaxis 

**Figure 6:**
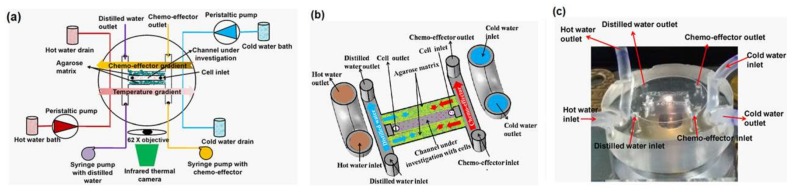
A microfluidic chip developed by Murugesan et al., which combines thermal and chemical gradients in a single chip to perform both thermotaxis and chemotaxis on *E. coli* bacteria at the same time. Reproduced from [16].

As an example of the thermotaxis of bacteria, Erickstad et al. fabricated a straight PDMS microchannel and generated a linear thermal gradient across it [82]. They injected a batch containing more than 10,000 *E. coli* and observed their thermotaxis. It took around 100 s for each batch to travel from the inlet to the outlet, and this makes their system a fast, high-throughput thermotaxis platform for *E. coli* bacteria. In another study, Murugesan and his collaborators fabricated a PDMS-agarose microfluidic device to analyze the thermotactic behavior of *E. coli* DH5α [83]. They added different concentrations of gold nanoparticles (AuNPs) to the solution to assess their effect on the thermotaxis of *E. coli*. They found out that the particles intervene in the thermotaxis process and inhibit cell migration. They reported the decreased level of adenosine triphosphate (ATP) as the reason for this failure. Apparently, gold nanoparticles reduce the F-type synthase activity of ATP. They have used the same platform for the chemotaxis of *E. coli*, as well. Their device configuration is shown in Figure 6.

### 3.2. Chemotaxis

Most of the microfluidic chips designed for chemotaxis study are made of PDMS and consist of separate inlet channels for chemoattractant, buffer and cells, merging into an experiment chamber. The dimensions of the device elements depend on whether the purpose of the study is quality or quantity. Diffusion as one of the two modes of mass transfer which is associated with random molecular motion within a fluid, is responsible for generating the chemical gradient in most of these devices, although geometric modifications to the device design can induce interlayer motion and enhance mixing. Nematodes, bacteria, leukocytes and sperm are common samples for chemotaxis experiments, as mentioned in the previous section.

#### 3.2.1. Microfluidics for Sperm Chemotaxis

**Figure 7:**
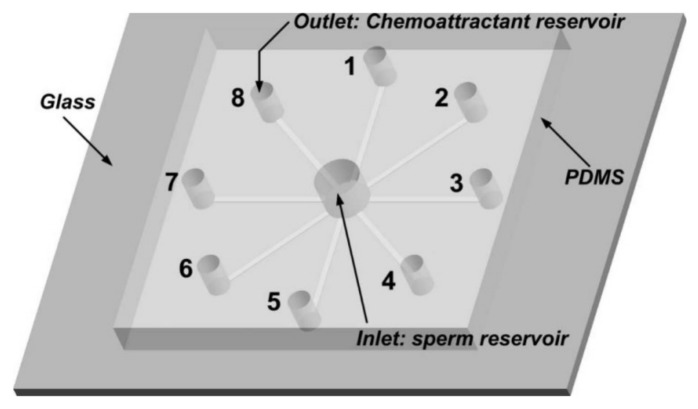
Microfluidic device fabricated by Ko et al. for chemotaxis on mouse sperm. Each of the circumferential reservoirs is filled with a sperm chemoeffector, and semen is injected in the center of the chip. Reprinted with permission from [84].

Ko et al. fabricated and tested a microfluidic platform to separate motile sperm from mouse semen [84]. Their device is PDMS on glass and consists of straight microchannels connecting the centric inlet to separate outlets in radial fashion (Figure 7).

**Figure 8:**
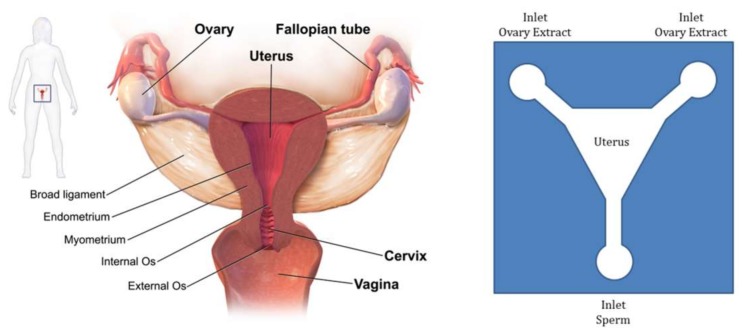
Picture on the left (© Lumen—Boundless Anatomy and Physiology) shows a schematic of the actual female reproductive system, while on the right is a microfluidic device concept in which two inlets are considered as the ovaries connected to the observation chamber (uterus) by microchannels (fallopian tubes) with an inlet at the bottom with which sperm samples are injected. The device is appropriate for the chemotaxis of sperm.

The travelling of sperm through female reproductive organs involves a biologically complicated phenomenon. Researchers have tried to replicate this whole journey on-chip to be able to analyze the exact behavior of sperm. For one of the attempts in this area, Xie et al. fabricated a single-layer microfluidic chip to replicate the mammalian female genital tract in order to test the motility and chemotaxis of sperm [85]. Figure 8 shows the schematics of the actual and the chip model of the female genital tract.

**Figure 9:**
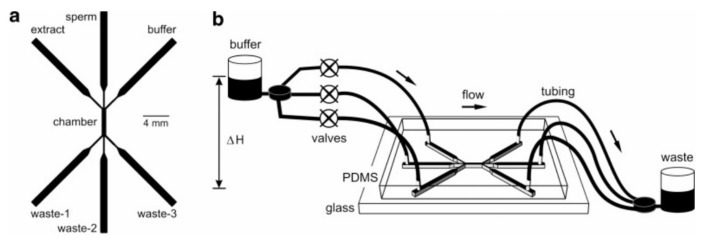
Schematic PDMS-glass microfluidic device and the experimental apparatus for sperm chemotaxis. Reprinted with permission from [86].

In another attempt, Koyama and the collaborators fabricated a PDMS-glass microfluidic device to mimic the chemotaxis of sperm toward ovaries [86]. They injected a mouse sperm sample from the middle inlet while a buffer and ovary extract flowed through side inlets and generated a uniform chemical gradient across the observation channel. They measured the drift of sperm quantitatively due to their chemotactic response toward the ovary extract while they swam along the channel. The schematic of their device along with the experimental apparatus is depicted in Figure 9.

#### 3.2.2. Microfluidics for Nematodes Chemotaxis

**Figure 10:**
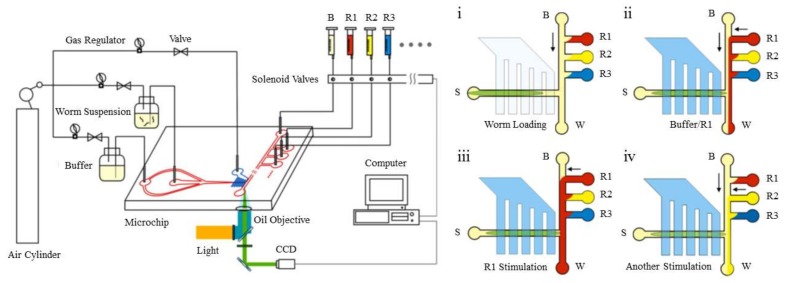
A sample platform for the chemotaxis study of *C. elegans*. In this study, the nematode is fixed by a vacuum, while its chemotactic response to different chemoattractants is analyzed. Reprinted from [77].

Nematodes are another group of organisms that are typically tested in chemotaxis experiments. Microfluidic designs for the chemotaxis of different types of nematodes are presented in this section. An example of a chemotaxis study on a *C. elegans* is shown in Figure 10. In this experiment done by Wang et al. [77], the nematode is held stationary by a vacuum, and its chemotactic response to three different chemicals is studied.

**Figure 11:**
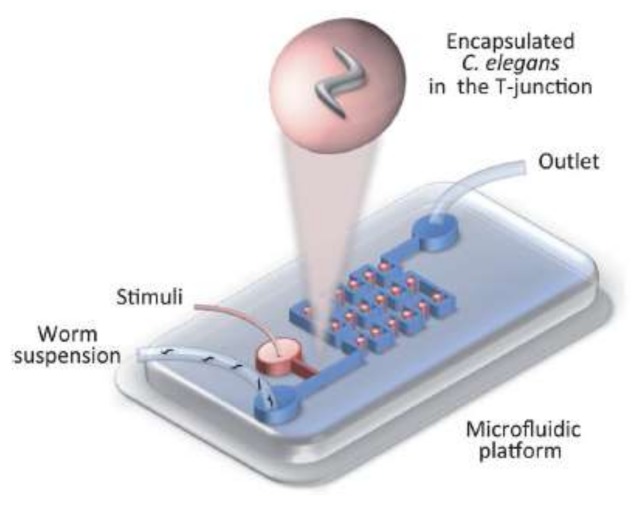
This schematic picture shows that cells and microorganisms can be trapped inside a moving droplet for biological experiments such as different types of taxis. Reproduced from [15].

The animal can also be trapped inside droplets and then be exposed to a thermal or chemical gradient to facilitate the study of its tactic behavior in a stationary situation (as shown schematically in Figure 11 [15]).

**Figure 12:**
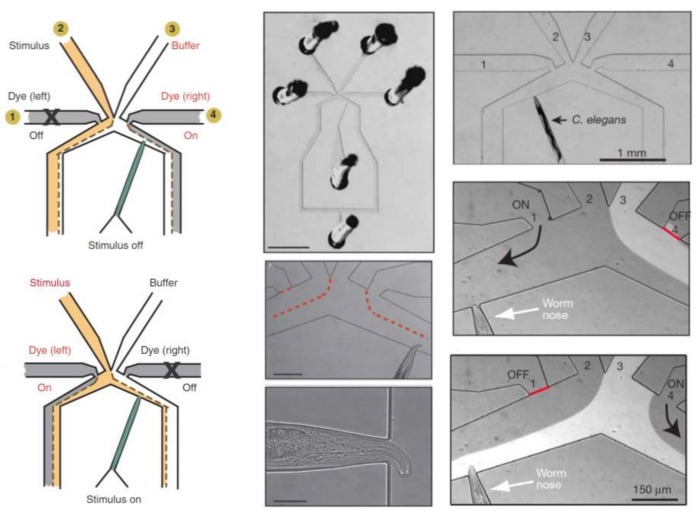
Microfluidic device developed by Chronis and his group for single-organism qualitative study of the chemotactic response of *C. elegans*. The device consists of inlets, one for the chemoeffector, one for the buffer and two side ones for dye. The device structure, the schematics of its functionality, as well as the orienting chemotactic response of the worm are shown in this figure. Reprinted by permission from [87,88].

In another approach, Chronis et al. designed and fabricated two devices (behavior chip and olfactory chip) to analyze the neuronal activity of *C. elegans* as a neuron model with different types of stimulation [87,88]. The olfactory chip is shown in Figure 12. This device consists of multiple inlets (one for stimulus, one for buffer, two for dye and one for sample) and two outlets and is made of PDMS. The advantage of this device is the capability of single cell analysis with more precision at the neuronal level.

**Figure 13:**
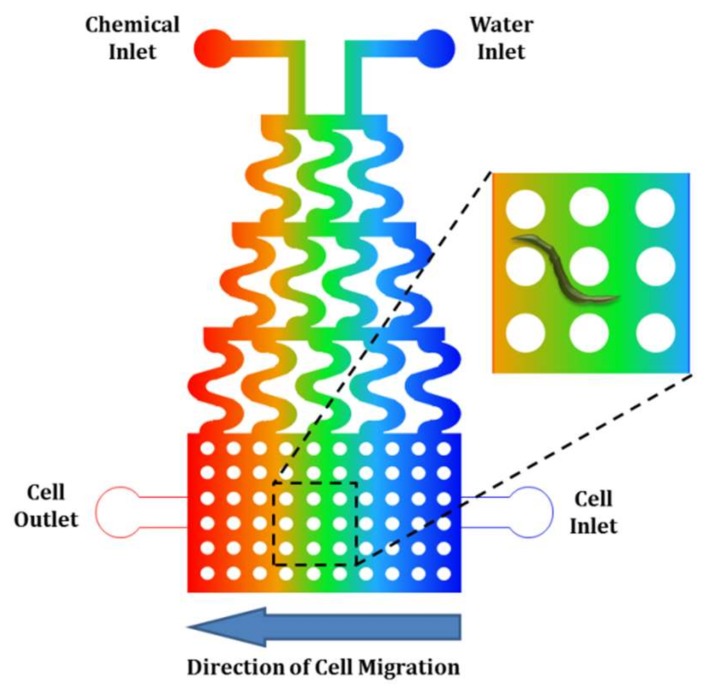
A microfluidic device developed for chemotaxis on *C. elegans*. In this device, the chemical gradient is generated by using serpentine channels. In this device, dirt and colloids inside soil are modeled with an array of microposts in the observation chamber. The idea is adopted from the microfluidic device developed by Hwang et al. [89].

Hwang et al. fabricated a microfluidic device on agar plates consisting of a comb section for the generation of a uniform chemical gradient and an observation chamber including an array of microposts [89]. The micropillars, cylinders of 300 μm diameter and 475 μm center-to-center distance, are fabricated to mimic the obstacles presented in soil. They could observe chemotactic behavior of *C. elegans* toward only nanomoles of NaCl, which is three orders of magnitude less than their chemotactic response level. They also observed the repellence effect of different concentrations of sodium dodecyl sulfate on *C. elegans*. Their device configuration is similar to what is shown in Figure 13.

**Figure 14:**
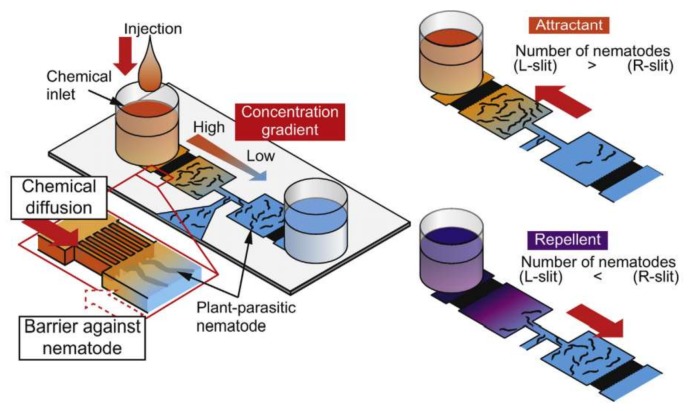
Microfluidic platform for chemotaxis of a nematode called *Meloidogyne incognita* or *M. incognita*. In this design, the experiment chambers are separated from the main inlets by barriers, which act like one-way valves. Reproduced with permission from [91].

*Steinernema feltiae* or *S. feltiae*, another soil-dwelling, entomopathogenic nematode, is the subject of a chemotaxis study by Stilwell and his coworkers [90]. Microfluidic devices for chemotaxis experiments can be fabricated easily and quickly as is described by them. They employed an inexpensive method and fabricated a one-inlet-two-outlet device out of two transparent films attached using an adhesive film. Another microfluidic platform was developed and implemented for the chemotaxis of nematodes by Hida et al. in 2015 [91]. They have studied the chemotactic behavior of *Meloidogyne incognita* (*M. incognita*) in response to potassium nitrate (KNO_3_). As is shown in Figure 14, their device consists of an inlet for the nematodes and two inlets for the chemicals. The chemical inlets are separated from the test chambers by arrays of narrow channels, which are too tight to let the nematodes pass toward the chemical inlets, hence working as barriers against nematodes. The same structure of barriers has also been used by Chikshi et al. [92].

#### 3.2.3. Microfluidics for Bacteria Chemotaxis

As an example of the chemotaxis of bacteria, Murugesan and his collaborators fabricated a microfluidic device to analyze the simultaneous effect of thermal and chemical gradients on the migration of *E. coli* [16]. As shown in Figure 6, there are side channels for hot and cold water to generate a longitudinal thermal gradient along the experiment chamber. A channel for DI water on the left and a channel for the chemoattractant (1 mM sorbitol or 1 mM NiSO_4_) on the right side of the device are responsible for the chemical gradient on the cells, which are swimming in a center channel sandwiched between two agarose matrices. They have concluded that in the presence of both gradients, the migration of *E. coli* is always initiated by the chemical gradient, but its rate and percentage is influenced by the local temperature.

**Figure 15:**
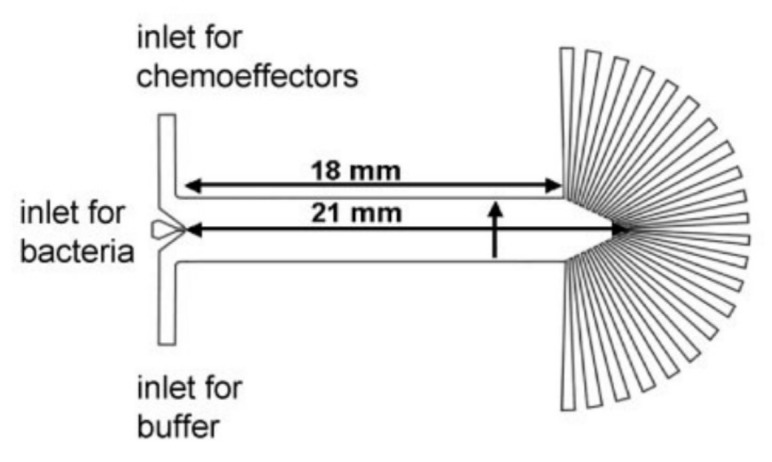
Schematics of a microfluidic device developed by Mao et al. for chemotaxis of *E. coli*. Inlet configurations induce a chemical concentration gradient across the channel and lead the bacteria to move toward the upper outlets, generating a spectral concentration of bacteria in the outlet channels. Reprinted with permission from [93].

In another attempt toward chemotaxis of *E. coli*, Mao et al. fabricated a microfluidic device, shown in Figure 15 [93]. They chose L-aspartate, L-serine, L-leucine and Ni^2+^ as chemoattractants and injected them through one of the inlets while a buffer solution was continuously injected through the other inlet. A chemotactic mutant *E. coli* sample was injected from the middle inlet and was forced to travel along the channel. The configuration of inlets provided a gradient of chemoattractant across the channel, which resulted in an unbalanced distribution of bacteria in radial output channels. Their device was used for the study of the chemotactic response of wildtype cells to the same chemoattractants, as well.

**Figure 16:**
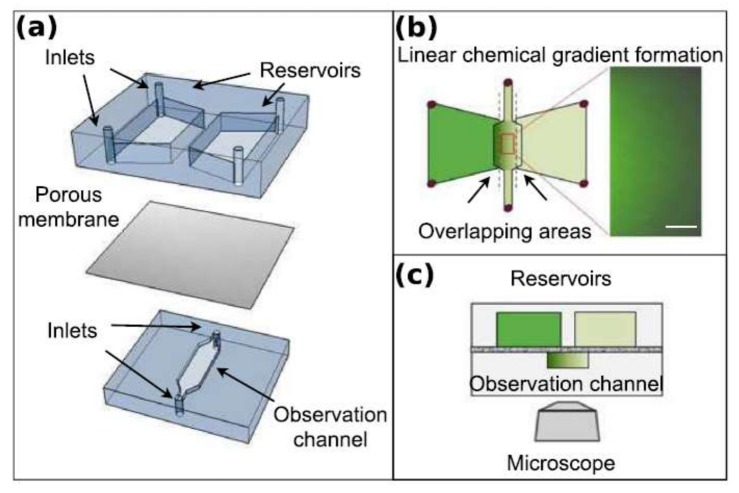
Schematic of the microfluidic device fabricated by Nagy et al. to perform chemotaxis experiments on *E. coli*. The lower level of the device consists of two separate reservoirs for the chemoeffector and the buffer, while on the upper level, the bacteria inlet and outlet are connected by a straight observation channel. The reservoirs have overlaps with the sides of the observation channel and are separated from it by an aluminum-oxide membrane. Depending on the pore size of the membrane, the chemical and the buffer are fed to opposite sides of the observation channel, generating a uniform chemical concentration gradient across the observation channel. The exact behavior of the batch of bacteria toward this concentration gradient is analyzed. Reproduced from [94].

Nagy et al. employed an interesting concept to generate a chemical concentration gradient across the observation channel to study the chemotactic response of *E. coli* [94]. They fabricated a double-layer microfluidics chip, which consists of two reservoirs on the top layer and the observation channel on the bottom layer. The reservoirs have an overlapping area with each side of the observation channel, while the top and bottom layers are separated by a porous aluminum-oxide membrane. The diffusion of the chemicals from each reservoir through the membrane into the observation channel produces the chemical concentration gradient necessary for the chemotaxis of *E. coli*. The schematic of their device is depicted in Figure 16.

**Figure 17:**
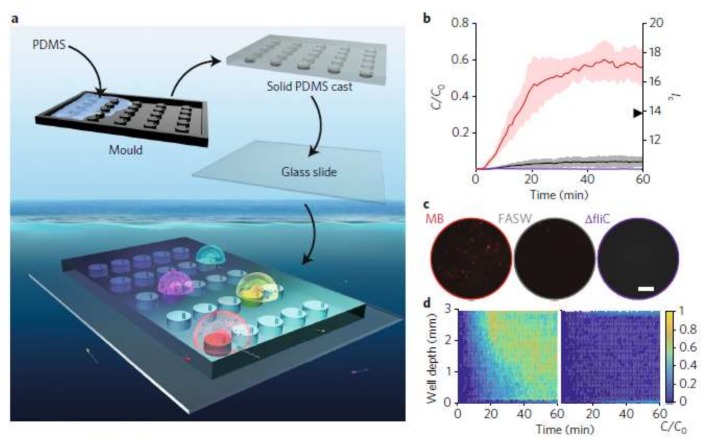
PDMS microfluidic chip for the chemotaxis study of marine microbials. Drops are placed on each port on top of separate reservoirs filled with different chemoeffectors and the amount of microbials attracted by chemicals and entering the reservoirs is measured. Reprinted with permission from [95].

In situ chemotaxis assay (ISCA) is a useful approach in quantitative chemotaxis studies. As an application related to chemotaxis of bacteria, Lambert et al. employed this technique for chemotaxis of marine microbes by induction of passive migration [95]. They 3D-printed the master mold and fabricated a PDMS-glass microplatform consisting of an array of wells, each enhanced with an independent port and enriched with a certain chemoattractant. The objective was inducing passive migration of microbes toward the ports into the wells and performing a quantitative study of in situ chemotaxis. Figure 17 shows the schematics of their device along with the results of their concentration experiments.

#### 3.2.4. Microfluidics for Chemotaxis of Cancer Cells

**Figure 18:**
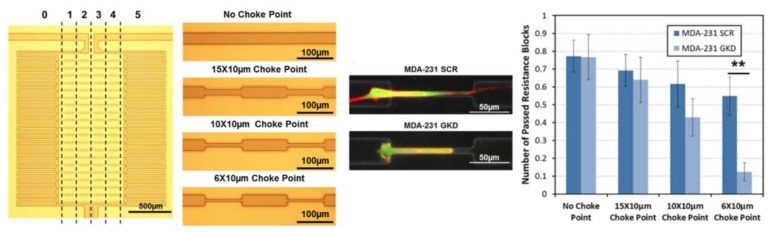
Microfluidic device for the chemotaxis of cancer cells and the simulation of lymphatic capillary geometry. The device consists of choke points with variations of widths from 6 μm (narrowest) to 30 μm (no choke point). Migration behavior of MDA-MB-231 cells through the choke points is analyzed. The differences of the migration patterns between scrambled control (SCR) cells and the p38γ knockdown (GKD) cells (** refers to *p* < 0.01). Reprinted with permission from [96].

Microfluidics has also contributed to the diagnosis and curing of cancer through the chemotaxis of cancer cells. A key factor in the heterogeneity of characteristics in cancer cells and the inception of metastasis is the ability of some of them to travel toward capillaries while others cannot. To analyze this behavior and address this chemotactic heterogeneity, Chen and his collaborators fabricated a single-layer PDMS-glass microfluidic chip consisting of an array of migration channels with choke points [96]. They examined two colonies of cells through the device, one chemotactic and the other non-chemotactic. Experiments were performed with three different dimensions of choke point widths (15, 10 and 6 μm) to mimic lymphatic capillary geometry and be able to explain cancer metastasis. Figure 18 shows a schematic of their device and a sample of cells passing through the choke points.

**Figure 19:**
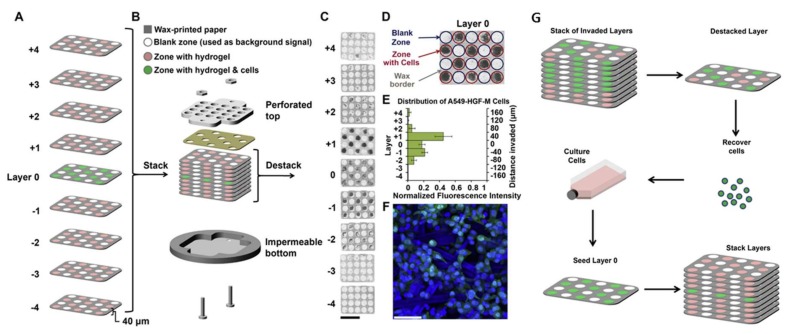
Multi-layer paper-based microfluidic device developed by Mosadegh et al. for chemotaxis of A549 cancer cells. Reprinted from [97].

Chemotaxis of cancer cells has been performed on paper-based microfluidic chips, as well. Mosadegh et al. developed a 3D paper-based microfluidic device comprised of a stack of wax-patterned papers impregnated with a cell-compatible hydrogel for the chemotactic assay of A549 cancer cells with oxygen and proteins (Figure 19) [97]. Three different types of A549 cancer cells have been used in their experiments, namely A549 (basal epithelial cells of a human alveolar adenocarcinoma), A549-HGF (A549 cells engineered to express hepatocyte growth factor (HGF) constitutively) and A549-HGF-M (a subgroup of the A549-HGF cells derived from a lung metastasis in a xenograft tumor). The quality of response of the cancer cells is analyzed by measuring the fluorescence intensity of different layers within the stack. The gradient of cells toward the chemoattractant is generated by adjusting the permeability of the papers to the chemoeffectors. Their work is the first experimental demonstration of chemotaxis of cancer cells with oxygen as the chemoattractant.

## 4. Microfluidics Inspired by Taxis

Actuation due to chemical or thermal gradients is meaningful even for a homogenous liquid inside a microchannel in the absence of living organisms. Mass transfer due to a surface tension gradient, which is called the Marangoni effect, is the basis of this motive. As is known, the surface tension of a given liquid is a function of temperature, chemical composition, surface roughness, electrical potential and so many other factors. Therefore, fluid migration can be realized by inducing a surface tension gradient by imposing a thermal or chemical gradient inside the medium. This phenomenon was first identified by James Thomson as “tears of wine” [98] and restudied by Carlo Marangoni in 1865 [99].

Moreover, the shear flow due to a surface tension gradient in conjunction with a solid substrate or another immiscible liquid causes fluidic interlayer motion. This phenomenon leads to fluid manipulation and mixing, droplet/bubble migration, sorting, merging, trapping and releasing and has applications in chemical sampling and reaction, biological assays, drug delivery and other in vitro and in situ experiments. In this context, droplets and bubbles are considered as isolated microcarriers of chemical samples and cells, which can be delivered to certain locations inside microplatforms with acceptable accuracy, in order to mimic fluid flow in organs.

### 4.1. Thermotaxis-Inspired Microfluidics

As was discussed in our previous review [100], a thermal gradient in microfluidic chips can be generated either by embedding metal microheaters or by using laser beams. The idea was first tested on drops of different liquids on solid substrates and then expanded to liquid-liquid closed and open microdevices in multiphase media. The Marangoni effect due to local heating induces inner shear flows inside the liquid, which under certain conditions may lead to thermocapillary instability [101,102,103,104,105,106,107]. Due to this inner motion, thermocapillarity is useful for fluid mixing [108,109,110], heating and evaporation [111].

Thermocapillary-based fluid migration is an interesting ongoing research area. Since a thermal gradient causes a surface tension gradient, a droplet of a given liquid can crawl on a solid substrate if it is under a thermal gradient. The theoretical formulation of such a problem has been developed under certain conditions [112,113,114]. Thermocapillary migration of a single drop on solid substrate has also been numerically solved with different methods, namely the level-set method [115] and the lattice Boltzmann method [116]. The phenomenon has also been shown experimentally by several research groups [117].

Thermal actuation of single drops on solid substrates was taken a step forward and applied to multiphase flows inside microchannels. Choudhuri and Sekhar formulated thermocapillary migration of a spherical drop inside another liquid using the Laplace equation for both hydrodynamic and heat transfer [118]. Naterer’s group took a closer look at thermal actuation by formulating the motion of the meniscus inside a microchannel [119,120]. Baird and Mohseni developed a theoretical model for migration velocity of a single drop inside a microchannel and assessed the contribution of different external forces, one of which being thermocapillarity [121]. Several benchmark problems of this nature have also been solved using different numerical methods, namely the finite element method (FEM) [122], finite difference method (FDM) [123,124,125], finite volume method (FVM) [126], lattice Boltzmann method (LBM) [127,128] and volume of fluid (VoF) [129], some of which have even taken droplet deformability into account [125,130].

**Figure 20:**
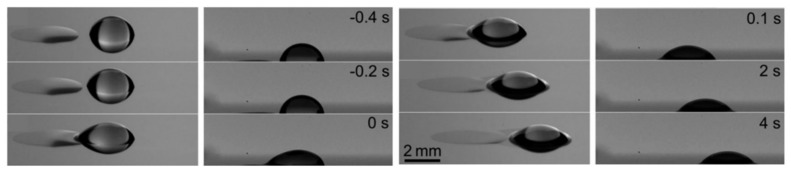
Migration of binary drops on a solid substrate by inducing thermocapillarity (analogous to thermotaxis). Reproduced from [117].

As has been described at the beginning of this section, the realization of thermocapillary actuation inspired by thermotaxis started on solid substrates using either embedded heaters [131,132,133,134] or laser beams [135,136,137,138,139]. As shown in Figure 20 [117], droplets crawl on the substrate when a thermal gradient (and consequently, a surface tension gradient) is induced.

**Figure 21:**
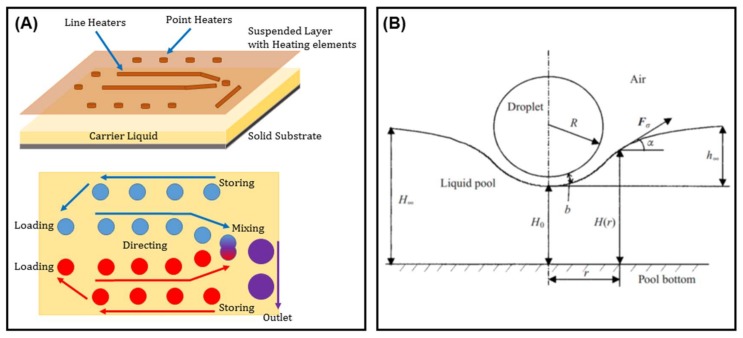
(**A**) Interplay of the Marangoni effect and gravity for the manipulation of levitated droplets on a carrier liquid using an indirect heat source. The idea was adopted [146]. (**B**) Introducing the concept of levitated droplets by Savino et al. Reproduced with permission of [147].

The same behavior has been seen when droplets or bubbles are flowing inside a carrier liquid in a closed microplatform. Not only was this concept used to direct emulsions along certain paths [140], but it also led to the fabrication of on-chip elements such as valves [141], pumps [142], traps [143], microcapacitors [144], micromirrors [145], etc. Later on, Basu realized thermocapillary actuation by fabricating arrays of microheater elements on a suspended plate on top of the carrier liquid bath (Figure 21) [146]. This work was inspired by the analytical discussion of the liquid surface deformation under a thermal gradient by Savino et al. [147].

**Figure 22:**
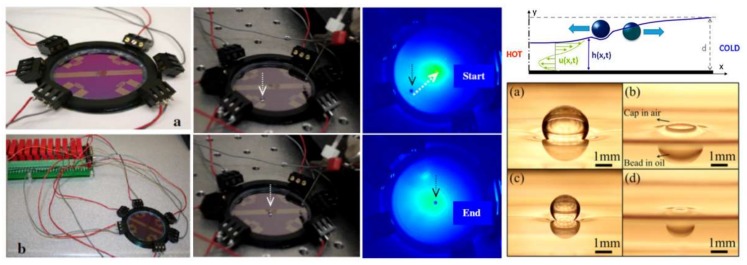
Analysis of the dual stable state of a droplet on an immiscible liquid film and its different response to heat sources inspired by positive and negative thermotaxis. Reprinted with permission from [150,153].

Cho’s group realized the thermotaxis of drops on-chip utilizing arrays of embedded Ti microheaters in the bottom of a shallow liquid bath [148]. They took advantage of the depression of the carrier liquid on the hot spot to manipulate levitated droplets and roll them toward heat [149,150]. They also discovered two different types of droplets corresponding to the carrier liquid (levitated and cap-bead), one of which is attracted to, while the other one is repelled by the hot spot [151,152,153], in a similar manner to living organisms (Figure 22).

### 4.2. Chemotaxis-Inspired Microfluidics

Chemical bonding strength between the liquid and the solid substrate influences the contact angle of the given liquid on the substrate [154,155]. Based on this phenomenon, the concepts of hydrophobic/hydrophilic and oleophobic/oleophilic surfaces are defined. Due to the contribution of the relative chemical composition to the contact angle, the manipulation of drops by inducing the Marangoni effect using a chemical gradient is a major microfluidic application inspired by chemotaxis. Analogous to thermocapillarity, the change of the contact angle by a chemical gradient may be called chemocapillarity (also called solutocapillarity).

The first apparent approach to realizing chemocapillarity on solid substrates was modifying their surfaces by coating them with certain chemicals. Some chemicals can make the surface hydrophobic, while some others enhance the spreading of liquids on substrates. For example, it is convenient to wash microchannels with ethanol to make the walls hydrophilic and enhance liquid migration before performing experiments. Treating surfaces with oxygen plasma is another way of decreasing their hydrophobicity. Chemical vapor deposition (CVD) is a frequently-used technique to coat a substrate with a thin layer of certain chemicals and thus to modify the wettability of their surfaces. The silanization of glass and Si substrates can also be useful for changing the surface characteristics and manipulating droplets [156]. Comprehensive reviews on the modification of surface wettability have been written, to which readers are referred for a more detailed discussion [157,158,159].

**Figure 23:**
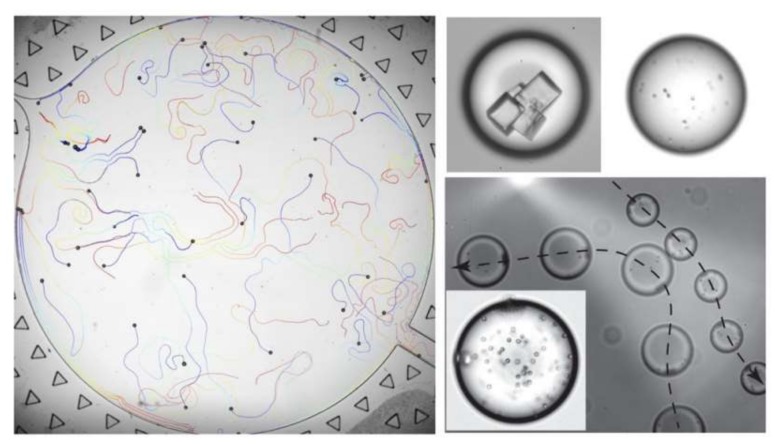
Chemotactic manipulation of water droplets encapsulated with crystals, cells and colloids in squalane oil with monoolein surfactant. Reprinted figure with permission from [163].

Another approach to reaching the desired contact angle is to mix the liquid with small concentrations of certain chemicals. Surfactants are specified for this purpose [160]. It has been proven that the form and strength of the Maragoni convection inside a liquid varies by changing its chemical composition [161,162]. Izri et al. have studied self-propelled groups of water droplets in squalane oil with monoolein surfactant [163]. They studied the effect of the Péclet number, the surface gradient of the solute and phoretic mobility on the characteristic autophoretic velocity and average migration velocity of the droplets. Their experiments proved that just like with chemotaxis, if the right pair of solutions and the proper surfactants with adequate concentrations are chosen, a self-propelled microfluidic system could be realized in which droplets work as carriers of cells, crystals or colloids. Figure 23 shows the color-coded migration path of a batch of droplets with time along with pictures of droplets encapsulating cells, crystals and colloids.

Zhao and Pumera fabricated a microcapsule that can find and remove pollutants from the environment using the chemocapillary technique [164]. In order to fabricate this capsule, they mixed polysulfone (PSF) with N,N’-dimethylformamide (DFM). It solidifies and forms a capsule as soon as it is introduced to water. Two interfaces are formed, one between the capsule and water and the other between the capsule and air. It is reported that the psf/water interface has a smaller pore size (130 nm) compared to that of the psf/air interface (20 μm). This asymmetry leads to the gradual releasing of DFM to the air. Since the surface tension between the psf and the water is stronger than that between the psf and the DFM, the capsule tends to migrate toward the pure water path and is repelled by the pollutants. To realize this migration, they produced an artificial pollutant and injected it in different locations in their microfluidic chip and observed the autonomous motion of the capsule to identify the source of pollution.

**Figure 24:**
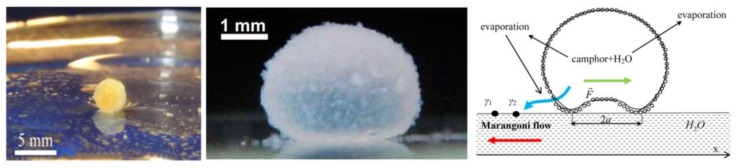
Self-propulsion microfluidic system inspired by chemotaxis for the manipulation of marbles on water. The left figure shows a marble filled with camphor, and the right figure shows a sample physical configuration of the problem. Reprinted with permission from [165]. The middle figure shows a moving marble containing 70% ethanol. Reprinted with permission from [166].

Bormashenko et al. realized self-propulsion of liquid marbles filled with either camphor or ethanol on a thin water surface owing to the Marangoni effect or, as they call it, solutocapillarity [165,166]. The marbles are coated with either lycopodium or fumed fluorosilica powder. Evaporated camphor from the marble is adsorbed by water asymmetrically, inducing a non-symmetric surface tension gradient between the marble and the water surface. This leads to oscillations in the velocity of the center of mass of the marble along with its angular velocity. Figure 24 shows the marble moving on top of the water surface.

A similar problem was designed and solved by Suzuno et al., involving maze solving using the chemotaxis concept [167]. They designed and fabricated a microfluidic chip in the form of a maze and filled it with an alkaline solution. A surface tension gradient was induced by a pH gradient throughout the channel from the inlet to the outlet by placing an acid-soaked hydrogel (hydrogels comprise a group of polymeric materials, whose hydrophilic structure enables them to hold large amounts of water in their three-dimensional networks [168]) at the outlet. This surface tension gradient can manipulate dye particles toward the outlet by inducing the Marangoni effect. They solved the same problem using thermocapillarity one year later [169].

## 5. Summary

This paper reviewed the microfluidic devices for the study of thermotaxis and chemotaxis and various design ideas in microfluidic devices inspired by these two phenomena. Numerous efforts have been made to analyze the thermo- and chemotactic responses of different types of cells, nematodes, bacteria, leukocytes and sperms. Microfluidics as a powerful framework has facilitated experiments using taxis phenomena. Novel concepts of microchips have been developed and implemented based on thermo- and chemotaxis to stimulate samples and study their behavioral response.

Regarding the sampling of microorganisms, devices may be designed for either high-throughput analysis or single-cell study. In high-throughput assays, a batch of microorganisms is injected in the device in each run, and their behavioral response to the stimuli is observed as a group. For a single-cell study, the quality of the response of one single microorganism to the stimulations is analyzed. A recently-developed idea that can facilitate this group of experiments involves encapsulating the microorganism inside a droplet and performing assays in multiphase media. This approach enables researchers to use active and passive microfluidic techniques to guide and control the samples through the device and immunizes the microorganisms from contamination. A single-cell assay may be performed in a stationary situation in which the microorganism is fixed at a certain location inside an experiment chamber. Various types of tweezers may be utilized for this purpose using geometrical, optical or fluidic confinements.

In terms of generating thermal and chemical gradients, various approaches have been used based on the type of taxis. Embedded or remote heaters such as counter-flow heat exchangers, resistive heaters and laser beams have been utilized to generate thermal gradients in thermotaxis microfluidic devices. Optical and resistive heaters, respectively, are less expensive to fabricate, easier to implement and more precise to control compared to heat exchangers. In order to generate chemical gradients on-chip in chemotaxis assays, diffusion-dominant transport mechanisms in laminar flows in microscale channels have been utilized. The easiest way to implement this concept is to inject the buffer and the chemoeffector from two separate inlets on the opposite sides of a channel and rely on diffusive mixing to generate the gradient perpendicular to the flow direction as the two chemicals flow side-by-side along the channel. Specific channel geometries such as serpentine shapes can be used to enhance mixing passively and create a more uniform chemical gradient. Multi-level microfluidic devices for generating chemical gradients have also been reported. In this approach, reservoirs for chemicals are fabricated on one layer and the channels on the other, while the two layers are separated by a porous membrane. The degree of complexity in fabrication increases from one to another in the aforementioned implementations.

Bio-inspired microfluidic devices have also been developed owing to the concepts of thermo- and chemotaxis. As is known, surface tension of any given liquid can be influenced by changes in the physical characteristics of its environment such as temperature, chemical composition, electrical charge and roughness of the surface. Inducing a surface tension gradient by imposing an external thermal or chemical gradient is used for fluid manipulation inside microchips for different purposes such as directing, sorting, merging, mixing, trapping and releasing of samples. Embedding resistive heaters, asymmetric chemical coating, chemical vapor deposition and plasma treatments are among the approaches that have been used to induce a surface tension gradient on solid substrates and multiphase flows. Adding ionic and non-ionic surfactants can also enhance the solutocapillary-based Marangoni effect. Table 1 categorizes microfluidic devices that have been used for or inspired by thermo- and chemotaxis.

Due to the progress in microfluidics, taxis on-chip can be developed further to facilitate in situ assays for anatomical analysis of organs, diagnosis of diseases and development of remedial treatments. The capability of other polymeric substrates with better physical characteristics and biocompatibility can be assessed for taxis experiments since most of the taxis devices so far are made of PDMS and glass. Taxis assays can also be extended to paper-based microfluidics to develop portable, point-of-care devices for clinical experiments. Enhancing fluidic mixing with geometrical and external elements, as well as using embedded resistive and remote optical microheaters can be helpful in fabricating novel microfluidic devices capable of generating more precise chemical and thermal gradients for taxis experiments. Moreover, open or closed microfluidic chips with modified interfaces and embedded resistive or optical heat sources for autonomous fluidic regulations can be further inspired by thermo- and chemotaxis. Thermo- and chemocapillarity can be used in droplet-based microfluidic devices for several applications such as biological assays and chemical reactions. Employing fast and inexpensive fabrication techniques with relatively straightforward designs, combining fluidic and embedded elements in biocompatible polymer-based or paper-based microfluidic chips and fabricating point-of-care diagnostic devices for taxis assays promises various future implementations.

## Figures and Tables

**Table 1:** Categories of microfluidic devices used for or inspired by thermo- and chemotaxis. HGF, hepatocyte growth factor.

		Object for Manipulation	Gradient Generation Method (Specsifications)	Application/Year/Reference
Microfluidics Used for Taxis	Thermotaxis	Nematodes (*C. elegans*)	Liquid Convection (15 °C–25 °C)	Single-Cell ASH Neuron Stimulation, 2011 [81]
		Sperm	Metal Sheet Conduction (34 °C–38 °C)	High-Throughput Motility, 2014 [80]
		Bacteria (*E. coli*)	Heat Exchanger (32 °C–37 °C)	High-Throughput Migration, 2017 [16]
			Heat Exchanger (35 °C–37 °C)	High-Throughput Congregation, 2012 [82]
			Heat Exchanger (32 °C–37 °C)	High-Throughput Migration with Au nanoparticles, 2016 [83]
		Cancer Cells (MDA-MB-231)	Heat Exchanger (26 °C–48 °C)	High-Throughput Viability/Activity Screening, 2008 [79]
	Chemotaxis	Nematodes (*C. elegans*)	Diffusion (Ethanol, NaCl)	Single-Cell ASER Neuron Stimulation, 2011 [76]
			Diffusion (Glycerol, SDS, Copper)	Single-Cell ASH Neuron Stimulation, 2011 [77]
			Diffusion (Glycerol, Calcium)	Single-Cell AVA and ASH Neurons Stimulation, 2007 [87]
			Diffusion (Calcium, Odor)	Single-Cell AWC, AIB and AIY Neurons Stimulation, 2007 [88]
			Diffusion (Glycerol)	Single-Cell ASH Neurons Stimulation, 2010 [92]
			Enhanced Diffusion (NaCl, SDS)	High-Throughput Migration, 2015 [89]
		Nematodes (*S. feltiae*)	Diffusion (Waxworm, Acetic Acid)	High-Throughput Migration, 2017 [90]
		Nematodes (*M. incognita*)	Diffusion (KNO_3_)	High-Throughput Migration, 2015 [91]
		Sperm	Diffusion (Acetylcholine)	High-Throughput Motility vs. Concentration, 2012 [84]
			Diffusion (Cumulus Cells)	High-Throughput In Vitro Fertilization, 2010 [85]
			Diffusion (Ovary Extract)	High-Throughput Motility, 2006 [86]
		Bacteria (*E. coli*)	Diffusion (Sorbitol, NiSO_4_)	High-Throughput Migration, 2017 [16]
			Diffusion (Aspartate, Serine, Leucine, Ni^2+^)	High-Throughput Migration, 2003 [93]
			Diffusion (Lysine, Arginine)	High-Throughput Migration and Quorum Sensing with *P. aeruginosa*, 2015 [94]
		Bacteria (*V. coralliilyticus*, *E. coli*)	Diffusion (Marine Broth, Methylaspartate)	High-Throughput Migration, 2017 [95]
		Cancer Cells (Skov3, A2780DK, C2C12, MDA-MB-231, PC3)	Diffusion (HGF)	Single-Cell Migration through Lymphatic Capillaries, 2015 [96]
		Cancer Cells (A549)	Diffusion (Oxygen)	High-Throughput Paper-based Migration, 2015 [97]
Microfluidics Inspired by Taxis	Thermotaxis-Inspired Microfluidics	Liquid on Solid	Embedded Resistive Heating	Thermocapillary Migration of Binary Drops, 2011 [117]
				Thermocapillary Router, Mixer and Valve, 2003 and 2010 [131,132]
				Thermocapillary Droplet Migration, 2008 [133]
				Thermocapillary Droplet Pumping, 1999 [142]
				Droplet-based Microcapacitor, 2004 [144]
				Droplet-based Micromirror, 2009 [145]
		Liquid in Liquid (Closed System)	Embedded Resistive Heating	Thermocapillary Droplet Migration, 2009 and 2010 [134]
			Remote Optical Heating	Droplet Switching, Sorting, Mixing, Merging and Splitting, 2007–2009 [135,136,137,138,140,141]
		Liquid on Liquid (Open System)	Embedded Resistive Heating	Droplet Storing, Routing, Trapping and Mixing, 2008 [146,148,149,150,151,152]
	Chemotaxis-Inspired Microfluidics	Substrate Surface Modification	Silanization (Alkyltrichlorosilane)	Fast Drop Migration on Modified Si Substrate, 2001 [156]
		Liquid-Liquid Manipulation	Surfactant (Squalane, Monoolein)	Migration of Carrier Water Droplets, 2014 [163]
			PH Alteration (PSf, DMF, SDS)	Self-propelled Capsules as Pollutant Sense-And-Act, 2014 [164]
			Gradual Release (Camphor, Ethanol)	Self-propelled Liquid Marbles on Thin Films, 2015 & 2017 [165,166]
			PH Gradient (Hexyldecanoic Acid)	Migration of Dye Particles in Maze, 2014 [167]
